# Essential and Non-Essential Elements in Razor Clams (*Solen marginatus*, Pulteney, 1799) from the Domitio Littoral in Campania (Southwestern Tyrrhenian Sea, Italy)

**DOI:** 10.3390/toxics10080452

**Published:** 2022-08-05

**Authors:** Mauro Esposito, Silvia Canzanella, Amalia Danese, Angela Pepe, Pasquale Gallo

**Affiliations:** 1 Istituto Zooprofilattico Sperimentale del Mezzogiorno, Department of Chemistry, Via Salute 2, 80055 Portici, Italy; 2 Istituto Zooprofilattico Sperimentale del Mezzogiorno, Centro di Referenza Nazionale per l’Analisi e Studio di Correlazione tra Ambiente, Animale e Uomo, Via Salute 2, 80055 Portici, Italy

**Keywords:** trace elements, metals, razor clam, Campania, risk assessment

## Abstract

The levels of essential (Cu, Cr, Co, Mn, Se, Zn) and non-essential (As, Be, Bi, Cd, Cs, Ga, Ni, Pb, Sr, Tl, U, V) trace elements were studied in razor clams (*Solen marginatus*) collected from the Tyrrhenian coast of Southern Italy at five selected sites along the Domitio littoral in the Campania region. The main objectives of this study were to assess the contamination status of these bivalve mollusks and to evaluate the risks to the environment and consumers due to metal contamination. The concentrations of 18 trace elements were determined after microwave-assisted mineralization and by inductively coupled plasma mass spectrometry (ICP-MS). Concentrations of the toxic elements Pb and Cd were below the maximum levels established by Commission Regulation (EC) 1881/2006, while higher average concentrations of arsenic were found at each of the five sites studied. Regarding the other trace elements, contamination levels followed the order: Zn > Sr > Mn > Cu > Se > Cr > V > Ni > Co > Ga > Cs > Be > U > Bi > Tl. No significant differences among the sites were found with regard to any of the trace elements analyzed, and element levels in razor clams did not reflect sediment contamination. The results demonstrated the substantial food safety of the razor clams in this area with respect to heavy metals but revealed a potential health risk due to arsenic contamination in all the areas sampled.

## 1. Introduction

Estuaries and coastal marine environments are vulnerable to a wide range of chemical pollutants from urban, agricultural and industrial activities. These pollutants affect the health of aquatic organisms and pose a potential risk for humans who consume them [1,2].

Among contaminants, some trace elements are of huge concern owing to their abundance, persistence and subsequent bioaccumulation in environmental matrices (air, water, sediment and biota). They have therefore been placed on the list of priorities of the European Water Framework Directive (2000/60/EC) [3,4,5].

Trace metals in the aquatic environment originate both from natural processes and from human activities. Natural processes mostly involve erosion, chemical rock weathering, volcanic activity or soil leaching, whereas anthropogenic sources are primarily urban surface runoff and agricultural, industrial and domestic activities [2,6].

To study the fate of metals in food webs, it is of great importance to assess the bioaccumulation of trace metals in marine organisms. Benthic organisms living in the sediments of coastal lagoons are exposed to pollution by metals and metalloids owing to their water-filtering capabilities, ability to concentrate chemicals and sedentary lifestyles [7,8]. As bivalves belong to the benthic taxa, they have been used as sentinels for assessing pollution levels in marine ecosystems since 1976 [9] and are considered good biomonitors of some environmentally relevant chemicals in marine and coastal areas around the world [10,11,12,13,14].

Among bivalves, the razor clam is a species of great interest as it is consumed by humans and has high commercial value [15]. The razor clam (*Solen marginatus*, Pulteney, 1799) is a member of the *Solenidae* family and lives buried in sand; its elongated shell enables it to penetrate easily into sediment [13,16]. This species has a wide geographic distribution, from Norway to North Africa including the Mediterranean Sea and the southeast and west coasts of England [12,15,17]. Razor clams can burrow into low intertidal and subtidal sediments to a depth of about 30 cm [18]. The rectangular and cylindrical bivalve shell of the clam is thin, narrow and smooth, allowing the animal to slip easily through the sand [13,18,19], in which it leaves only a small keyhole-shaped hole.

Since the razor clam lives in contact with marine sediments and, like most bivalve species, filters large amounts of water, it tends to accumulate organic and inorganic pollutants from food, water and particulate matter; these xenobiotics may then enter the food chain, posing a risk to consumers [13,20,21,22,23]. The extent of bioaccumulation is closely related to geographic location; the species, size and feeding behavior of the animal; and the solubility and lipophilicity of the chemicals involved and their persistence in the environment [24].

While many of the elements found in fish and shellfish, such as selenium, iron, zinc and copper, are essential to humans at low concentrations, non-essential elements such as cadmium, lead and mercury and metalloids such as arsenic are toxic even at low levels of exposure [24]. Therefore, monitoring and controlling the levels of metals and metalloids in benthic organisms is important in order to assess pollution levels in vulnerable areas and to protect consumer health.

Currently, the European Union regulation classifies three metals—Pb, Cd and Hg—as potentially hazardous for human consumption [25], and the World Health Organization has established a provisional tolerance of weekly intake of these elements to protect public health [26]. Although razor clams have great commercial value [15,16], there are still too few data on their distribution, abundance, bioaccumulation and consumption-related risks in an area of great environmental interest, namely the Domitio littoral in the Campania region.

An ecotoxicological study carried out by Balassone et al. [27] recorded some critical values of chemical and biological parameters along the coast of Falerno-Domitio, mainly due to the presence of hazardous substances (Cr and V) originating from extensive anthropic activities. Moreover, the results of their study confirmed a relationship between the accumulation of metals and the moderate toxicity of some near-shore marine sediments [27]. Sediments are essential to the functioning of aquatic ecosystems and are extremely important to the food web; however, they can provide a reservoir for contaminants, which can promote bioaccumulation and biomagnification across trophic levels [28,29,30]. 

Considering the pollution from the above area and the fact that razor clams live in close contact with marine sediments and filter large amounts of water [13], we aimed to determine the concentrations of trace elements (As, Be, Bi, Cd, Co, Cr, Cs, Cu, Ga, Mn, Ni, Pb, Se, Sr, Tl, U, V and Zn) in razor clams farmed along the coast of the Domitio littoral in order to: (i) assess the presence of metals for which the European Union Commission has established a limit to ensure consumer safety; (ii) characterize the overall risk of dietary exposure of the adult population to toxic elements through the consumption of razor clams sampled in a potentially contaminated environment; (iii) verify the use of razor clams as sentinel species for monitoring the status of the marine environment in the vicinity of a highly contaminated area.

## 2. Materials and Methods

### 2.1. Study Area

The present study was carried out on the Domitian coast, a wide coastal strip on the Tyrrhenian Sea that extends for about 50 km in the northern part of the Campania region, from Capo Miseno to the mouth of the Garigliano River [27,29]. In 1998, the Domitian coast was declared a Site of National Interest (N.I.S.) by the Italian government, owing to its potentially high risk of contamination [27,29]. Since 2013, competence for the reclamation work of this area has been transferred from national to regional by Italian authorities, as established by the Ministerial Decree of 11 January 2013. The Domitian coast where the razor clams analyzed in this study were collected, is adjacent to the so-called “Land of Fires”, a well-known area where illegal waste disposal, including the practice of burning or burial, has taken place for years [31]. In addition, the Domitian coastal area hosts three important hydrographic basins: the Garigliano River, the Volturno River and the Regi Lagni basin, which contribute to undermining the ecological integrity of the marine and coastal area [32,33].

The Volturno River is heavily polluted by discharges from zootechnical facilities, chemical factories, urban sewage and agricultural runoff [34]. In addition, the release of environmental pollutants resulting from incineration and illegal waste dumping has degraded the quality of soil, water and air and affected the waters and sediments of the Domitio littoral, into which the river flows [32].

Like the Volturno, the Regi Lagni river basin, which consists of a network of channels flowing from the plain north of Naples to the Tyrrhenian Sea, is in a completely neglected state and is heavily polluted as a result of urbanization, industrialization (especially the chemical industry), intensive agriculture and buffalo farming [33].

Other rivers also flow into the sea along the Domitian coast, such as the Savone River, the Agnena Canal, the Camaldoli Canal and the last stretch of the basin of Lake Patria. Some of these waterways are heavily polluted by civil and industrial effluents and the uncontrolled discharge of waste [33].

### 2.2. Sampling Location

**Figure 1:**
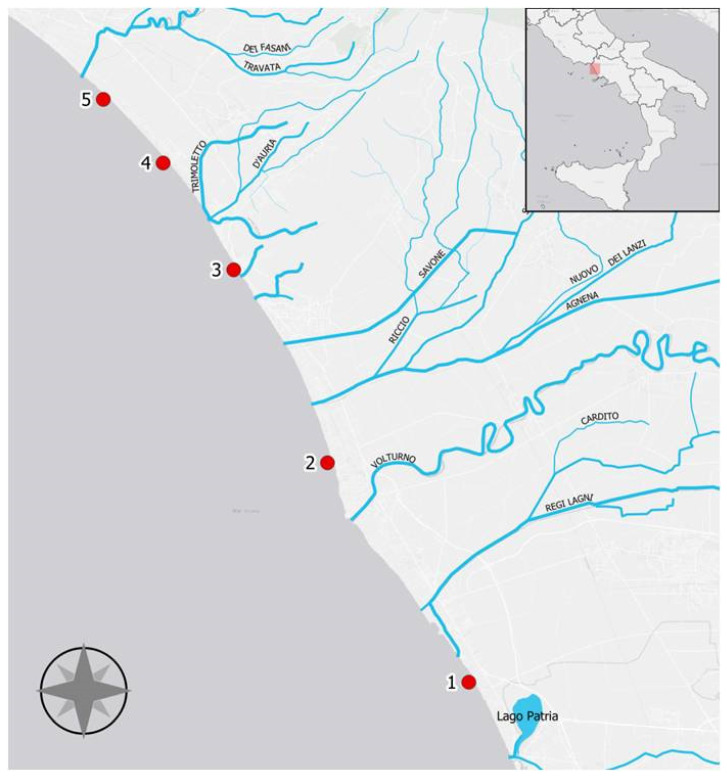
Map of the collection sites along the Domitio littoral (Campania region, Italy): (1) Castelvolturno-Ischitella; (2) Castel Volturno-Lavapiatti; (3) Mondragone; (4) Cellole; (5) Sessa Aurunca.

As shown in Figure 1, five different coastal stations were selected; their GPS coordinates are: Castelvolturno-Ischitella (40°57′1.73″ N, 13°59′50.80″ E), Castelvolturno-Lavapiatti (41°2.56.22″ N, 13°54′41.61″ E) Mondragone (41°8′8.49″ N, 13°51′14.64″ E); Cellole (41°11′0.83″ N, 13°48′39.62″ E); Sessa Aurunca (41°12′43.09″ N, 13°46′28.94″ E). 

### 2.3. Sample Collection and Preparation

A total of 26 samples of razor clams (*S. marginatus*), each sample consisting of 40 specimens, were collected by hand from sandy beaches at 5 sampling stations along the Domitian coast between 2018 and 2021, in order to quantify the accumulation of essential and non-essential trace elements in the soft tissues. The mollusks collected were packed in sterile plastic bags and sent to the laboratory of the Istituto Zooprofilattico Sperimentale del Mezzogiorno. 

Before analysis, the shell length and soft tissue weight of the specimens were measured: The average shell length ranged from 6 to 10 cm, and the average edible weight ranged from 3.3 to 6.0 g. Samples were then washed with ultrapure water to remove mud or adhering particles, and the edible portion (soft tissue) was excised from the shells with a plastic knife. The soft tissue samples were homogenized, frozen and stored at −20 °C until analysis. 

Chemical analyses were performed in accordance with the Commission Regulations establishing analytical methods for the official control of the levels of chemical contaminants in food [35]. In addition, the performances, including sensitivity, of all the analytical techniques used in this study were carefully checked during the validation of the methods and proved to comply with the requirements of the above-mentioned regulations.

### 2.4. Analysis of Trace Elements

For trace element analysis, 0.75 g of each homogenized sample was placed in a glass test tube and subjected to acid mineralization with 5.0 mL of 70% nitric acid, 2.5 mL of 30% hydrogen peroxide and 2.5 mL of ultrapure water using a Milestone Ultrawave microwave digestion system (FKW, Torre Boldone, Italy). The test tubes were cooled at room temperature, and the samples were quantitatively recovered by filtration through paper disks in 25 mL Class A volumetric flasks and then made up to 25 mL with ultrapure water. 

An inductively coupled plasma mass spectrometer (ICP-MS, PerkinElmer NexION 3500X) equipped with, a concentric nebulizer baffled cyclonic spray chamber (Glass Expansion, Inc., West Melbourne, Australia) and a quartz torch with quartz injector tube (2 mm internal diameter) was used to quantify trace elements. The instrumental parameters were as follows: radio frequency power: 1200 watts; plasma gas (argon, Ar) flow rate: 15 L min^−1^; nebulizer gas (Ar) flow rate: 0.97 L min^−1^; sample flush: 60 s; sample flush speed: 32 rpm; read delay: 20 s; read delay and analysis speed: 20 rpm; wash: 45 s; wash speed: 32 rpm; dwell time: 50 ms; sweeps/reading: 20. As an internal standard, rhodium at a concentration of 200 ng mL^−1^ was added to the standard and sample solutions by means of on-line mixing.

The concentrations of the trace elements were measured in whole soft tissues. Two replicates of each sample were analyzed, and concentrations were evaluated as the mean of both measurements, with results expressed in mg kg^−1^ wet weight (ww).

In the mineralized solution, quantitative determination was performed according to the standard addition method by means of calibration curves at four spiking levels: for U and Tl at 0.001–0.005–0.020–0.10 ng/mL; for Co, Cr, Mo, Ni and V at 0.1–0.5–2.0–10 ng/mL; for Cu and Mn at 0.5–2.5–10–50 ng/mL, and for Zn and Sr at 4–20–80–400 ng/mL. The correlation coefficients (R^2^) of the standard calibration curves for all trace elements were always higher than 0.99, showing an excellent linear relationship across the selected concentration ranges.

The limits of quantification (LOQs) of the method were calculated as 10 times the standard deviations of the signals from 20 blanks after mineralization. 

### 2.5. Quality Assurance and Quality Control Tests

Quality assurance and quality control (QA/QC) of the methods were monitored by analyzing procedural blanks, duplicate samples and standard solutions. Chemical blank determinations were performed periodically along with each sample batch to check for reagent purity and possible laboratory contamination. Certified Reference Materials NIST-SRM 2976 (Mussel Tissue—Community Bureau of Reference) were used to validate the analytical method and evaluate precision and accuracy. The accuracy of the method was also assessed with proficiency testing: the reported z-scores were within ±2, indicating that the values obtained were not statistically different from the values assigned.

### 2.6. Statistical Analysis 

All data were expressed as arithmetic means and standard deviations (SD) and were statistically analyzed using GraphPad Prism 9 (GraphPad Software, Inc., San Diego, CA, USA). Since the variables were not normally distributed (Kolmogorov–Smirnov test), a nonparametric test was performed. To evaluate differences among the five sampling sites, the Mann–Whitney test for independent samples was used, and a p-value of less than 0.05 was considered statistically significant.

## 3. Results and Discussion

The mean concentrations of the trace elements (As, Be, Bi, Cd, Co, Cr, Cs, Cu, Ga, Mn, Ni, Pb, Se, Sr, Tl, U, V and Zn) in the edible parts of razor clams collected along the Domitian coast in the Campania region are summarized in Table 1. The concentration of these elements was expressed in mg kg^−1^ wet weight (ww) in order to compare the values obtained in this study with the maximum levels established by EU legislation. The Commission Regulation [25] and subsequent amendments set maximum levels for Cd (1.0 mg kg^−1^ ww), Pb (1.5 mg kg^−1^ ww) and Hg (0.5 mg kg^−1^ ww) in bivalve mollusks, while no EU limits are imposed on other metals in this type of food. 

Cd and Pb belong to the group of non-essential and toxic metals and have no known function in biochemical processes [36]. The concentrations of Cd and Pb in the samples of razor clams ranged from 0.013 to 0.045 mg kg^−1^ and from 0.025 to 0.20 mg kg^−1^, respectively, i.e., well below the permissible limits.

To the best of our knowledge, there are very few data in the literature on trace element levels in *S. marginatus*; moreover, when reported, they are expressed in mg kg^−1^ dry weight [12], which does not allow for comparison with the data obtained in this study. However, it was possible to make a comparison with other bivalve species (*C. gallina*, *R. philippinarum*, *R. decussatus* and *M. galloprovincialis*) used as bioindicators of environmental pollution that were collected both in the same areas as those of this study and in other countries. Specifically, we compared the concentrations of elements in mollusks that, like razor clams, live in the sand. The concentration ranges of Cd and Pb in our study were similar to those measured in *C. gallina* in Veneto [37], Marche [38] and Abruzzo [39] and to those detected in *R. philippinarum* in Veneto [40] and *R. decussatus* in the lagoon of Sardinia [24] (Table 2). In contrast, we found lower concentrations than those measured by Breda et al. [41] in *R. philippinarum* in Portugal (Table 2). The mean concentrations of Cd (0.025 ± 0.0066) mg kg^−1^ and Pb (0.095 ± 0.043) mg kg^−1^ in *S. marginatus* were also comparable with those found in *M. galloprovincialis* collected in the same areas [42,43], indicating a similar capacity for bioaccumulation in both species (Table 2).

Unlike Cd and Pb, As had a high mean concentration (1.6 ± 0.23) mg kg^−1^, which may be due to the ability of bivalves to accumulate large amounts of this element. In its inorganic forms, As is toxic, but mollusks generally have high levels of total arsenic (tAs), mostly in the form of organoarsenic (arsenobetaine) compounds [24,44,45]. Arsenobetaine is neither toxic nor carcinogenic in mammals, but the frequency of seafood consumption may pose potential risks to human health [24,46]. Indeed, the risks of As bioaccumulation due to shellfish consumption have also been highlighted in recent studies in Portuguese systems, namely the Ria de Aveiro lagoon [41,47], the Óbidos lagoon [14,48] and the Tagus estuary [46].

The range of As concentrations (1.26–2.09) mg kg^−1^ in this study was similar to the values found in *C. gallina* clams from the Marche region [38] but much lower than those found in *R. decussatus* in Sardinia [24] and in *R. philippinarum* from Portugal [41,46]. Regarding the essential elements, the bioaccumulation of the metals detected in the samples followed this order: Zn (13.6 ± 2.4) mg kg^−1^ > Mn (3.3 ± 1.7) mg kg^−1^ > Cu (1.2 ± 0.35) mg kg^−1^ > Se (0.51 ± 0.17) mg kg^−1^ > Cr (0.34 ± 0.28) mg kg^−1^ > Co (0.11 ± 0.034) mg kg^−1^.

Comparison with literature data on other bivalve species (Table 2) showed that both the average value and the concentration range of Ni (0.046–0.78) mg kg^−1^ were lower than those found in *C. gallina* in the Marche region [38] and in *R. philippinarum* in Portugal [46,48] and similar to those found in *R. decussatus* in Sardinia [24]. In contrast, the range of Cr concentrations measured in our study (0.056–1.3) mg kg^−1^ was higher than in *R. philippnarum* from Portugal [41] and in *R. decussatus* from Sardinia [24] but lower than in *C. Gallina* from Marche [38] and in *R. philippinarum* from Portugal [46]. 

With the exception of V (0.31 ± 0.18) mg kg^−1^ and Sr (8.8 ± 1.9) mg kg^−1^, both of which had high mean values, the remaining elements (Be, Bi, Cs, Ga, Ni, Tl, and U) showed low concentrations: (0.020 ± 0.0068) mg kg^−1^ for Be, (0.0084 ± 0.0072) mg kg^−1^ for Bi, (0.022 ± 0.011) mg kg^−1^ for Cs, (0.055 ± 0.029) mg kg^−1^ for Ga, (0.23 ± 0.015) mg kg^−1^ for Ni, (0.0037 ± 0.0026) mg kg^−1^ for Tl, and (0.016 ± 0.0073) mg kg^−1^ for U. 

The differences in metal concentrations among the sampling sites did not show a clear spatial trend. Similar to the razor clams, the concentrations of the individual elements in the sediments reported by Balassone et al. [27] are similar along the Domitian coast, including Castel Volturno, Mondragone and Baia Domizia (near Cellole). Moreover, in agreement with other studies [12,46,47], single concentrations of elements in clams were not directly correlated with, or proportional to, single element concentrations in sediments. 

The lack of correlation can be explained by the presence of different chemical forms of elements in the sediments, depending on sediment particle size, composition, pH and oxidative conditions [49]. As a result, each of these metal forms has a different remobilization potential and bioavailability, which affects their bioaccumulation in the clams [41,46,49].

### Health Risk Assessment

The risk to human health from food consumption is usually assessed by comparing the estimated weekly intake (EWI) of the product with the provisional tolerable weekly intake (PTWI). The PTWI is defined as the estimated amount of a substance in food or drinking water, expressed on the basis of body weight (mg kg^−1^ or μg kg^−1^ body weight), that can be ingested weekly over a lifetime without constituting an appreciable health risk [26]. The PTWI is set by the Joint Expert Committee on Food Additives [50] of the Food and Agriculture Organization/World Health Organization (FAO/WHO) and Food Standards Australia and New Zealand (FSANZ). The average annual consumption of mollusks in Italy is 5.37 kg/capita/year, which corresponds to 103 g/capita/week [51]. However, since razor clams are a minor species in comparison with other traditional shellfish and are collected on a small scale in Italy, the health risk calculated on the basis of the average consumption of mollusks might be overestimated. In the absence of data on the average national consumption of razor clams, this study used PTWI as a benchmark for calculating trace element levels associated with the consumption of razor clams [12,24,41,46,48]. The amount of clam soft tissues that a 70 kg adult must consume in a week to exceed the PTWI is shown in Table 3. This value was determined by dividing the PTWI of a 70 kg adult by the concentration of a given element in clams. 

The results indicate a potential health risk due to As contamination in all areas studied, as <1 kg of shelled razor clams is sufficient to exceed the PTWI [12,46]. 

It is important to note that the results presented here refer to total As content in clams, whereas the JECFA and FSANZ limits and the PTWI were established for inorganic As only. However, as mentioned above, arsenic is bioaccumulated in seafood mainly in the form of nontoxic organic forms, while toxic inorganic forms are present only in a small percentage [50]. Element speciation analysis would allow for more accurate quantification of the risks engendered by this metalloid [12]. 

Regarding the other elements (Cd, Pb, Ni and Cu), humans run no risk of excessive intake unless they consume very large amounts of the product) (Table 3).

## 4. Conclusions

Razor clams, which have high nutritional and economic value, are suitable bioindicators for use in microbiological and chemical environmental monitoring in the marine environment. Our results showed that toxic trace elements in the clam samples tested did not exceed the maximum levels set by EU legislation. Moreover, the contamination levels of potentially toxic elements were below the tolerance limits for human consumption (PTWI) set by the International Scientific Expert Committee (JEFCA, FSANZ). While these results indicate the substantial food safety of razor clams in this area with respect to heavy metals, they also raise concerns regarding potential As contamination in all the areas surveyed since the consumption of 0.58 kg per week of this species is sufficient to exceed the PTWI. Therefore, although the Domitian coast is an area of national interest, owing to its high pollution potential, the razor clams collected from this coast can be considered safe for consumers and are not affected by the pollutants released by the rivers that flow into the sea in this area. 

## Figures and Tables

**Table 1:** Trace element concentrations in the razor clam expressed in mg kg^−1^ ww (mean ± standard deviation).

	Site 1 CI ^(1)^	Site 2 CL ^(2)^	Site 3 MO ^(3)^	Site 4 CE ^(4)^	Site 5 SA ^(5)^	Total Mean ± SD
	Mean ± SD	Mean ± SD	Mean ± SD	Mean ± SD	Mean ± SD	
As	1.6 ± 0.26	1.5 ± 0.22	1.6 ± 0.18	1.6 ± 0.20	1.8 ± 0.35	1.6 ± 0.23
Be	0.024 ± 0.0040	0.019 ± 0.0068	0.022 ± 0.0059	0.019 ± 0.012	0.016 ± 0.0026	0.020 ± 0.0068
Bi	0.0076 ± 0.0065	0.011 ± 0.010	0.0078 ± 0.0021	0.0045 ± 0.0040	0.011 ± 0.010	0.0084 ± 0.0072
Cd	0.026 ± 0.0054	0.023 ± 0.0038	0.023 ± 0.0045	0.024 ± 0.0092	0.030 ± 0.013	0.025 ± 0.0066
Co	0.12 ± 0.037	0.11 ± 0.027	0.13 ± 0.036	0.13 ± 0.039	0.088 ± 0.041	0.11 ± 0.034
Cr	0.37 ± 0.25	0.43 ± 0.41	0.24 ± 0.14	0.46 ± 0.28	0.13 ± 0.10	0.34 ± 0.28
Cs	0.025 ± 0.011	0.020 ± 0.0092	0.022 ± 0.0065	0.030 ± 0.019	0.014 ± 0.0035	0.022 ± 0.011
Cu	1.4 ± 0.38	1.3 ± 0.32	1.2 ± 0.32	1.1 ± 0.27	1.0 ± 0.20	1.2 ± 0.35
Ga	0.068 ± 0.034	0.053 ± 0.032	0.053 ± 0.013	0.059 ± 0.042	0.030 ± 0.0078	0.055 ± 0.029
Mn	4.2 ± 2.3	3.1 ± 1.7	3.6 ± 1.5	3.0 ± 1.5	2.0 ± 0.88	3.3 ± 1.7
Ni	0.25 ± 0.16	0.27 ± 0.23	0.17 ± 0.067	0.25 ± 0.070	0.14 ± 0.11	0.23 ± 0.015
Pb	0.13 ± 0.052	0.078 ± 0.028	0.090 ± 0.024	0.12 ± 0.057	0.059 ± 0.030	0.095 ± 0.043
Se	0.56 ± 0.13	0.50 ± 0.20	0.50 ± 0.20	0.53 ± 0.24	0.45 ± 0.12	0.51 ± 0.17
Sr	9.6 ± 1.3	8.6 ± 1.4	9.5 ± 3.1	6.9 ± 1.3	7.8 ± 0.79	8.8 ± 1.9
Tl	0.0047 ± 0.0027	0.0035 ± 0.0028	0.0036 ± 0.0031	0.0026 ± 0.0022	0.0040 ± 0.0026	0.0037 ± 0.0026
U	0.018 ± 0.0089	0.014 ± 0.0067	0.017 ± 0.0041	0.015 ± 0.012	0.013 ± 0.0032	0.016 ± 0.0073
V	0.38 ± 0.18	0.26 ± 0.17	0.31 ± 0.14	0.50 ± 0.23	0.14 ± 0.074	0.31 ± 0.18
Zn	15.1 ± 1.3	13.2 ± 1.7	13.7 ± 3.8	12.4 ± 1.6	12.6 ± 2.6	13.6 ± 2.4

^(1)^ CI: Castelvolturno–Ischitella; ^(2)^ CL: Castelvolturno- Lavapiatti; ^(3)^ MO: Mondragone; ^(4)^ CE: Cellole; ^(5)^ SA: Sessa Aurunca.

**Table 2:** Trace element concentrations (mg kg^−1^ ww) in bivalve mollusks from different locations.

Location	As	Cd	Pb	Ni	Cr	Reference
	Mean (Range)	Mean (Range)	Mean (Range)	Mean (Range)	Mean (Range)	
*M. galloprovincialis*						
Campania region (Italy)		0.05 (0.02–0.13)	0.15 (0.03–0.40)			[42]
Campania region (Italy)		0.022 (0.002–0.045)				[43]
*C. gallina*						
Veneto region (Italy)		0.06 (0.01–0.48)	0.10 (0.03–0.47)			[37]
Abruzzi region (Italy)		(0.075–0.14)	(0.036–0.14)			[39]
Marche region (Italy)	2.41 (1.61–3.74)	0.078 (0.023–0.18)	0.096 (0.013–0.36)	0.80 (0.35–6.14)	0.68 (0.086–10.6)	[38]
*R. philippinarum*						
Veneto region (Italy)		(0.052–0.099)	(0.16–0.24)			[40]
Portugal	6.38 (2.42–21.8)	0.078 (0.06–0.10)	1.26 (0.45–2.78)	1.79 (1.25–2.58)	3.63 (2.00–6.38)	[46]
Portugal	5.82 (4.80–6.84)	0.17 (0.16–0.18)	0.83 (0.78–0.90)	0.04 (0.03–0.05)	0.26 (0.23–0.29)	[41]
Portugal	(1.3–1.53)		(0.21–0.29)	(0.45–1.53)	(1.05–1.26)	[48]
*R. decussatus*						
Sardinia region (Italy)	5.4 (1.6–9.6)	0.044 (0.010–0.079)	0.17 (0.059–0.30)	0.63 (0.40–0.95)	0.15 (0.052–0.31)	[24]

**Table 3:** PTWI (provisional tolerable week intake) and amount of clams that a 70 kg adult needs to consume to exceed PTWI for each element and site.

		As	Cd	Pb	Ni	Cu
PTWI (mg Kg^−1^ week^−1^ WW)	JECFA ^(1)^	0.015 ^(3)^	0.007	0.025	0.035	3.5
	FSANZ ^(2)^	0.015 ^(3)^	0.007	0.025		
PTWI (mg week^−1^) for 70 Kg adult person		1.05	0.49	1.75	2.45	245
Amount of clams (Kg of WW) to be consumed to exceed PTWI						
Site 1 CI		0.66	18.8	13.5	9.8	175
Site 2 CL		0.70	21.3	22.4	9.1	188
Site 3 MO		0.66	21.3	19.4	14.4	204
Site 4 SA		0.58	16.3	29.7	17.5	245
Site 5 CE		0.66	20.4	14.6	9.8	223

^(1)^ PTWI set by the Joint FAO/WHO Expert Committee on Food Additives (JECFA). ^(2)^ PTWI set by the Food Standards Australia and New Zealand (FSANZ). ^(3)^ PTWI values for As are referred to inorganic As.

## Data Availability

All data will be made available upon reasonable request.
